# Accuracy of Real-Time PK-Guided Melphalan Dosing in Achieving Target Exposure in Myeloma Patients Undergoing Autologous Transplant

**DOI:** 10.3390/pharmaceutics18080924

**Published:** 2026-07-27

**Authors:** Kyeongmin Kim, Yizhen Guo, Min Hai, Kasey Hill, Nicole Abbott, Matias Eugenio Sanchez, Chukwuemeka Uzoka, Ana Maria Avila Rodriguez, John G. Quigley, Nadim Mahmud, Damiano Rondelli, Douglas W. Sborov, Donald Harvey, Donald J. Irby, Ajay K. Nooka, Madhav V. Dhodapkar, Jonathan L. Kaufman, Nisha S. Joseph, Sagar Lonial, Pritesh Patel, Mitch A. Phelps, Craig C. Hofmeister, Karen Sweiss

**Affiliations:** 1 Department of Pharmacy Practice, University of Illinois Chicago, Chicago, IL 60607, USA; 2 Division of Pharmaceutics and Pharmacology, The Ohio State University, Columbus, OH 43210, USAphelps.32@osu.edu (M.A.P.); 3 Comprehensive Cancer Center, The Ohio State University, Columbus, OH 43210, USA; 4 Division of Hematology and Oncology, University of Illinois Chicago, Chicago, IL 60607, USA; 5 Huntsman Cancer Institute, University of Utah, Salt Lake City, UT 84112, USA; 6 Department of Hematology and Medical Oncology, Winship Cancer Institute of Emory University, Atlanta, GA 30322, USA

**Keywords:** melphalan, autologous transplant, pharmacokinetics, PK-guided dosing, nonlinear mixed effects modeling

## Abstract

**Background**: High-dose melphalan 140–200 mg/m^2^ (HDM) with autologous stem cell transplant (ASCT) is standard first-line treatment in multiple myeloma (MM), yet standard BSA-based dosing results in wide variation in systemic exposure (AUC). We developed a pharmacokinetic (PK)-guided dosing strategy using a 100 mg/m^2^ first dose, enabling real-time PK assessment and individualized adjustment for the second dose. We report final results of Phase A of our multi-center Phase 1 trial (NCT04483206, MyMel) evaluating feasibility and accuracy of this personalized approach. **Methods**: Patients received melphalan 100 mg/m^2^ on Day −3, and seven PK samples were collected and shipped overnight for LC-MS/MS analysis. Real-time AUC estimation using noncompartmental analysis (NCA) guided Day −1 dosing to achieve pre-specified AUC targets (13.5 or 14.5 mg × h/L). For comparison, post hoc Bayesian estimation using a nonlinear mixed effects (NLME) model was performed. Sparse sampling designs were evaluated using NONMEM. **Results**: All 20 patients successfully received PK-guided dosing, with Day −1 doses determined within 48 h. PK-guided dosing reduced AUC variability (CV 4.20–5.62%), with 19 of 20 achieving AUCs within ±10% of the target, compared to what would have been achieved by BSA dosing (CV 9.74–15.34%). NLME improved accuracy, particularly in patients with missing samples, and maintained performance using only four PK time points. **Conclusions**: This study demonstrates that PK-guided dosing is accurate and feasible with HDM-ASCT. NLME enhances accuracy and enables simplified sampling. Phase B will identify maximum tolerated systemic exposure of seven additional AUC cohorts using the NLME model and a four-sample design.

## 1. Introduction

High-dose melphalan (HDM) followed by autologous stem cell transplantation (ASCT) remains standard frontline treatment for multiple myeloma (MM) based on randomized trials demonstrating significant improvement in progression-free survival (PFS) [1,2,3,4,5,6]. Unfortunately, outcomes after ASCT can be highly variable, with up to 40% of patients failing to achieve a complete remission (CR), and up to 30% of patients experiencing severe melphalan-associated oral mucositis [7,8,9,10]. This is in part attributed to significant interpatient variability in melphalan pharmacokinetics (PK) and exposure (area under the plasma drug concentration–time curve, AUC) [11,12].

Despite decades of use, HDM dosing has remained largely unchanged and is administered as a fixed dose based on body surface area (BSA), either as a single or 2-day split dose of 200 mg/m^2^ for fit patients, or 140 mg/m^2^ for patients with significant renal and/or cardiac dysfunction, older age, or frailty [13]. In prior studies in MM and lymphoma, BSA-based HDM dosing with these empiric dose adjustments has been reported to result in up to five-fold variation in melphalan plasma exposure [11,12,14,15,16,17,18,19]. Importantly, this exposure variability has been associated with variability in toxicity and treatment efficacy. Higher melphalan exposure (above the median of 12.84 mg × h/L) has been associated with improved overall survival in patients undergoing HDM-ASCT [14,15]. At the same time, higher exposure has been linked to increased risk of severe oral mucositis, febrile neutropenia, extended hospitalization, and delayed hematologic recovery [11,14,15,18,19,20]. Cardiac toxicities, including supraventricular tachycardia and atrial fibrillation, are also observed following standard BSA-based HDM dosing [21,22,23,24], although their associations with exposure have not been formally evaluated. Although the clinical relevance of melphalan exposure is well recognized, current dosing strategies fail to address and correct the interpatient variability in exposure.

To address this limitation, several groups, including ours, have proposed PK-guided dose individualization strategies for HDM, with emphasis on modeling approaches to predict melphalan exposure, but all based on single-center retrospective or observational data [19,25,26,27]. Only a small number of studies have gone beyond the prediction to prospectively implement real-time PK-guided dosing and formally evaluate the accuracy of target AUC attainment in clinical practice [28,29,30,31]. Success in target AUC attainment has varied (Table S1). Notably, Shah et al., in 2019 reported successful attainment of target AUC (13.5 ± 1 mg × h/L) in 13 out of 15 patients by adjusting the second dose [29]. While encouraging, this study was limited by a small sample size and a single-center design, restricting generalizability. Furthermore, although clinical variables, such as fat-free mass and renal function, have been shown to impact melphalan AUC, a priori dose adjustment using a population PK model incorporating these covariates was insufficient to accurately predict AUC and be used alone to guide dosing [31]. Most importantly, despite recognition of exposure–outcomes relationships in retrospective studies, an optimal AUC target for HDM in MM has not yet been established, as a therapeutic threshold maximizing efficacy and minimizing toxicity has not been fully elucidated. Furthermore, studies that have explored exposure-response outcomes have now spanned a time frame of over a decade and a half, and with evolving clinical practice (i.e., use of MRD testing in the clinic, change in IMWG response criteria assessment), there is a need for more contemporary studies that implement current standard of care.

We previously demonstrated feasibility and rapid turnaround time of a real-time clinical PK testing strategy that would enable PK-guided melphalan dosing [19]. Building on this, we designed a multi-center, Phase 1 clinical trial (NCT04483206, ‘MyMel’) to systematically evaluate a PK-guided melphalan dosing strategy and identify the recommended Phase 2 AUC exposure based on toxicity and response. Our ultimate goal is to address what has been lacking with prior melphalan PK-guided studies by (1) testing the feasibility and logistics of real-time PK-guided dosing across two institutions, with samples being shipped to a third institution, (2) prospectively demonstrating accurate AUC target attainment, (3) using a structured Phase 1 exposure-escalation framework with uniform and current consensus-based response and toxicity assessments, and (4) executing an exposure-response evaluation to identify the target melphalan exposure for a Phase 2 trial.

To our knowledge, this is the first Phase 1 melphalan AUC escalation trial using PK-guided dosing in a multi-center setting. Here we report the final results of the Phase A portion of our multi-center Phase 1 trial aimed at demonstrating feasibility and success of achieving AUC targets in the first two cohorts.

## 2. Materials and Methods

### 2.1. Clinical Trial Design and Patients

This is a Phase 1 clinical trial intended to test a total of 9 AUC targets and determine the maximum tolerated systemic exposure (MTSE). The study uses PK-guided dose adjustment to achieve a pre-specified total target AUC (i.e., combined AUC for 2 days of chemotherapy). This study was approved by the Institutional Review Board (IRB) of the Winship Cancer Institute of Emory University (IRB# 2025P011158) and the University of Illinois Chicago (IRB# 2020-0816). Table S2 summarizes Phase A and B of the trial. This study was reported in accordance with the TREND (Transparent Reporting of Evaluations with Nonrandomized Designs) guidelines [32].

Phase A was a pre-planned run-in required by the FDA to demonstrate the feasibility and accuracy of achieving the two lowest pre-specified AUC targets of 13.5 mg × h/L (Cohort 1) and 14.5 mg × h/L (Cohort 2). Successful completion of Phase A was required prior to enrolling into Phase B, which proceeds to escalate AUC cohorts to identify the MTSE using a 5 + 5 design. This will then be followed by a 20-patient expansion of the MTSE cohort. In our study, we will be collecting and assessing multiple endpoints (Table S3): the primary endpoint for efficacy is Day +90 MRD-negative complete response (MRD-negative CR) rates; and the primary endpoints for toxicity are clinically significant mucositis, tachyarrhythmia, and delayed engraftment based on protocol-defined criteria.

The trial is being conducted at two institutions, Emory University (Emory) and University of Illinois Chicago (UIC), with liquid chromatography-tandem mass spectrometry (LC-MS/MS) analysis being performed at a third institution (The Ohio State University, OSU). To enroll into Phase A, adult patients ≥18 years old with a confirmed diagnosis of MM per International Myeloma Working Group (IMWG) [33] criteria were required to have received two or more prior lines of therapy [34]. We were required by the FDA to enroll patients in the second-line setting and with successful completion of Phase A, they could permit enrollment of all patients, including those in the first-line setting. Additional eligibility criteria included creatinine clearance >40 mL/min, as determined by an estimated glomerular filtration rate (eGFR) using the Cockroft–Gault equation, which represents the currently accepted standard at both institutions to calculate eGFR, and clinical suitability for standard ASCT with 200 mg/m^2^ HDM, as determined by the treating physician. Written informed consent was obtained from all patients prior to enrollment.

Study participants were identified and screened for eligibility by investigators and research staff at the participating sites through review of clinic schedules and medical records. Eligible patients were approached by their treating physicians or clinical care teams and informed about the study, including its potential risks, benefits, and alternative treatment options. Participants were recruited from Winship Cancer Institute at Emory University, the University of Illinois Chicago, and through physician referrals to the participating sites. Eligible participants were enrolled sequentially into escalating AUC (exposure) cohorts according to the study’s exposure-escalation design.

Each patient received an initial dose of melphalan 100 mg/m^2^ on Day −3 prior to transplant, with BSA calculated using actual body weight. Melphalan infusions were administered by nurses trained in blood and marrow transplantation care and/or experienced Phase 1 clinical research nurses. Plasma PK samples were collected for AUC estimation and a PK-guided personalized dose was administered on Day −1 to achieve the assigned total target AUC. During Phase A, PK-guided Day −1 dose was determined using noncompartmental analysis (NCA), as required by the FDA, to confirm achievement of the pre-specified AUC targets.

### 2.2. Real-Time Personalized High-Dose Melphalan Workflow

**Figure 1:**
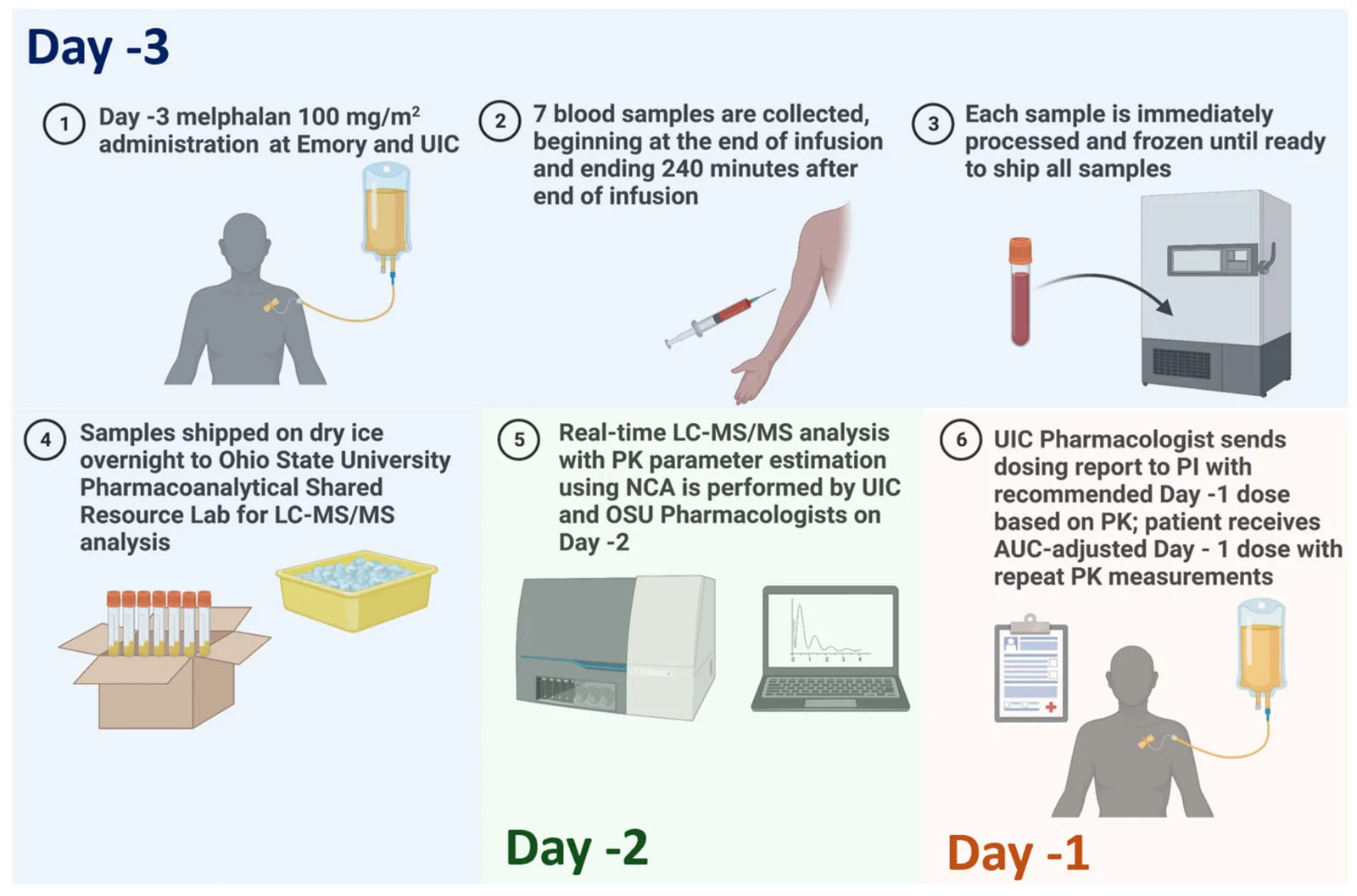
MyMel real-time workflow for sample collection, processing, and shipping, PK analysis, and Day −1 dose recommendation. Created in BioRender. Patel, P. (2026) https://BioRender.com/0h3tkvv (accessed on 28 June 2026).

The Phase A real-time workflow is illustrated in Figure 1. Patients were admitted either to the Phase 1 unit or the inpatient hospital and received melphalan at a dose of 100 mg/m^2^ on Day −3. All doses were administered based upon actual body weight, regardless of body mass index. Melphalan was reconstituted in 250 mL normal saline and infused over 30 min. Melphalan infusion was planned to complete within 45–60 min of drug reconstitution to prevent potential drug degradation when exceeding this time. Of note, interim analysis of our procedures was performed, and a decision was made to include additional information on the requisition sheets: time of drug reconstitution, time of sample processing, and time samples entered −80 freezer. This was done to ensure that variability in the timing of these steps could be factored into our PK data analyses and interpretations.

PK sampling, processing, and shipping were conducted using a standard workflow based on previously established methods [19]. A standard protocol for melphalan administration and real-time sample collection was implemented at both clinical sites to ensure uniformity, precision in timing, and optimal quality of samples. For sampling, a total of seven blood samples were collected at 0, 5, 15, 30, 75, 150, and 240 min after the completion of 30 min melphalan infusion (corresponding to nominal times of 0.5, 0.58, 0.75, 1, 1.75, 3, and 4.5 h after start of infusion) on Day −3. Samples were promptly processed, placed on dry ice, and shipped out to OSU Pharmacoanalytical Shared Resource (PhASR) for melphalan concentration measurements by LC-MS/MS. The same sampling schema was repeated on Day −1 with samples processed, frozen, and shipped in batches to PhASR. Standard PK requisition sheets were completed for each dose and shipped with samples.

A web application for a real-time melphalan dose adjustment was developed in R using the Shiny Package (v1.6.0) and deployed for online access via a password-restricted interface (i.e., the MyMel Shiny App). The application was utilized to support PK-guided dosing by integrating de-identified clinical information, dosing details, and melphalan concentration–time data in real time. The integrated data were exported into a .csv file for subsequent NCA and nonlinear mixed effects (NLME) analysis. Once NCA results were obtained, NCA-estimated melphalan drug clearance (CL) on Day −3 was entered into the MyMel Shiny App to calculate the recommended melphalan dose on Day −1 to achieve the target exposure. The principal investigator (PI) then made the final decision for the Day −1 dose to be administered.

### 2.3. Melphalan PK Parameter Estimation and Dose Adjustment

NCA was performed using Phoenix WinNonlin (Certara USA, Princeton, NJ, USA) with a .csv file generated by the MyMel Shiny App to estimate melphalan PK parameters for each patient. Day −3 CL and AUC (area under the curve from time of dosing extrapolated to infinity, i.e., AUC_0-inf_) were estimated using the linear up/log down method. These estimates were then used to calculate the personalized Day −1 dose required to achieve the pre-specified total AUC targets (13.5 mg × h/L for Cohort 1 and 14.5 mg × h/L for Cohort 2), defined as the combined AUC from both dosing days, using the relationship AUC = Dose/CL based on the linear PK of melphalan [25]. After Day −1 PK samples were collected and analyzed, actual total AUC (i.e., sum of Day −3 and Day −1 AUCs) was calculated to assess the performance of the PK-guided dose adjustment in achieving the pre-specified AUC target.

To compare observed total AUCs under PK-guided dosing with those that would have been achieved under standard BSA-based dosing, AUC under standard dosing was simulated. Administration of 200 mg/m^2^ was assumed, delivered as 100 mg/m^2^ on Day −3 and 100 mg/m^2^ on Day −1, and total AUC was calculated using the NCA-estimated CL from Day −3 and Day −1.

### 2.4. Post Hoc Evaluation of NLME-Based Dose Adjustment

To retrospectively evaluate whether an NLME population PK approach could achieve similar AUC targeting performance as the NCA-based method, a post hoc analysis was conducted. A previously published melphalan population PK model (NLME model) was applied to the current study cohort to estimate individual melphalan PK parameters (NLME method) [11]. Briefly, a two-compartment model with a single, first-order elimination term described melphalan PK, and creatinine clearance, hematocrit, fat-free mass, race, and sex were retained as covariates. Values for all fixed-effect parameters (THETA) from the model were fixed, and individual empirical Bayes estimates of CL on Day −3 and Day −1 were obtained using NONMEM version 7.5.0 under ADVAN6 with first-order conditional estimation with interaction (FOCEI). The NLME-estimated Day −3 CL was used to calculate the corresponding Day −3 AUC and to derive Day −1 dose under the NLME method. Day −1 AUC that would have resulted was calculated using the NLME-estimated Day −1 CL. Total AUC under the NLME method (${Total\;AUC}_{NLME}$) was then used to assess AUC target attainment and was compared with the total AUC observed under NCA-based PK-guided dosing. Post-processing of NONMEM outputs, AUC and dose calculations, and data summarization were performed in R version 4.3.2.

### 2.5. Sparse Sampling Design Optimization

Sparse sampling designs were evaluated to reduce the number of melphalan PK samples and shorten the sampling duration. Using the $DESIGN option in NONMEM (version 7.5.0), candidate designs with 7, 5, and 4 samples were optimized within pre-specified sampling windows, with the earliest time point at the end of the infusion (0.5 h after the start of infusion) and the latest constrained to 4.5, 4, 3.5, or 3 h after the start of infusion. From this analysis, a minimum of four time points was identified as sufficient to obtain parameter estimates with accuracy and precision similar to the original 7-sample scheme (see Supplementary Materials Methods and Results).

To assess the performance of the optimized sampling scheme, the observed PK data from the 20 Phase A patients were used to construct two 4-sample test designs (M4-1 and M4-2) by selecting observed time points closest to the optimized sampling times. CL estimates and ${Total\;AUC}_{NLME}$ obtained using these sparse sampling designs were generated as described in the previous section and compared against those from the full 7-sample design.

## 3. Results

### 3.1. Baseline Characteristics

A total of 20 patients were enrolled into the Phase A portion of this study, 10 each in Cohorts 1 and 2. Baseline characteristics of all patients in Phase A are summarized in Table 1. Median age is 64 (49–75) years, with 14 (70%) male and 9 (45%) Black patients.

### 3.2. Feasibility of Real-Time Melphalan Dose Adjustment

All 20 patients received standard melphalan 100 mg/m^2^ on Day −3, with a total of 140 planned blood samples (100%) collected on Day −3. All shipped Day −3 samples were received in suitable frozen condition, and all LC-MS/MS analyses with NCA estimation and Day −1 dose determination were completed within 12 h of receiving samples. For three patients (ID 8, 11, and 18), samples were delayed by one day during shipment, and bioanalytical results were not available until later in the morning on Day −1. These patients received their Day −1 dose later in the day, resulting in only 4 (*n* = 1) or 5 (*n* = 2) of 7 PK samples collected on Day −1. As a result, 133 (95%) of the 140 planned samples were collected on Day −1. All 20 patients received an adjusted dose on Day −1 based on the Day −3 NCA results.

### 3.3. NCA Observations and Dose Adjustments

**Figure 2:**
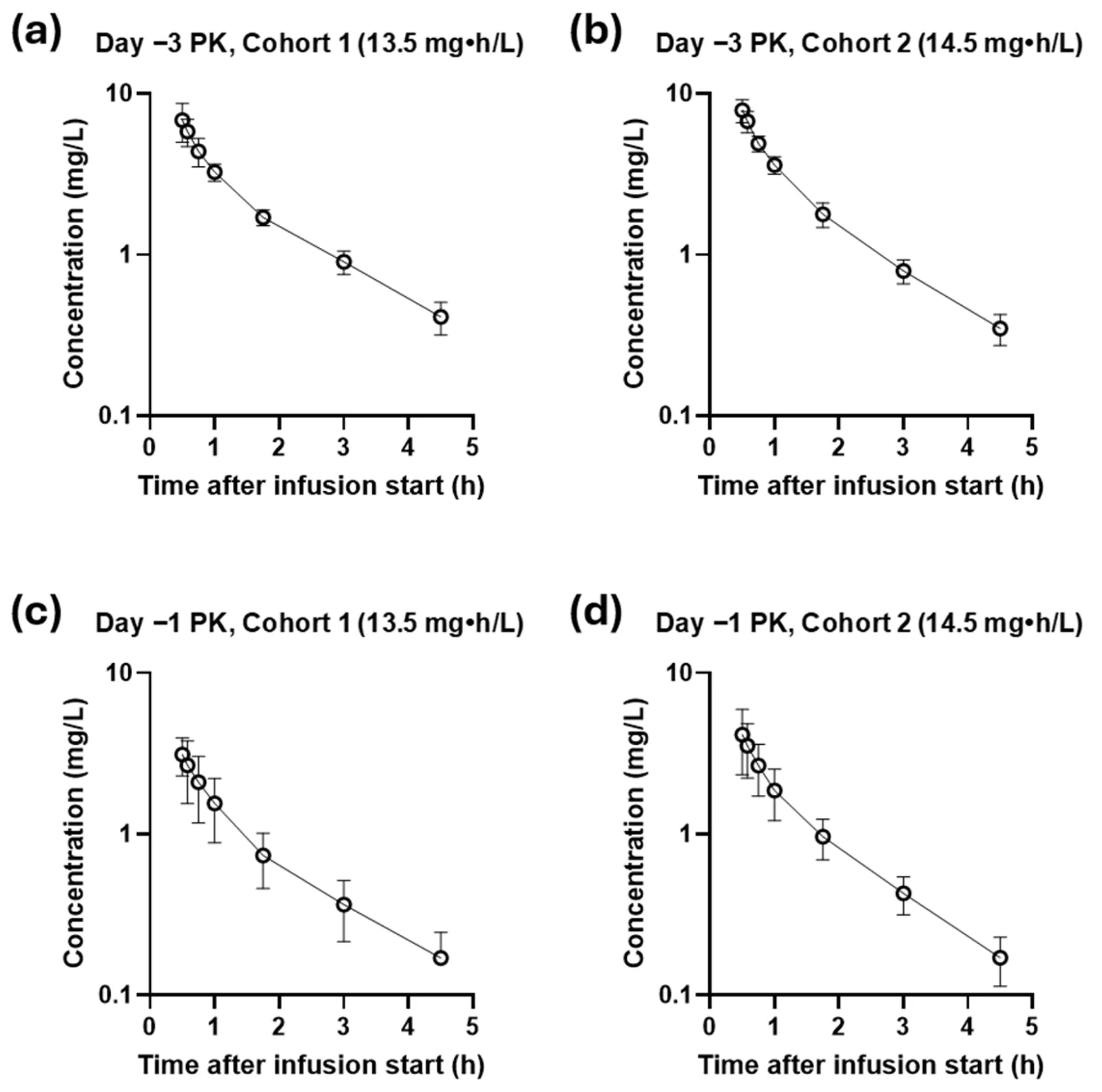
Melphalan plasma concentration vs. time (**a**,**b**) on Day −3 and (**c**,**d**) Day −1. Data shown as mean ± SD. The x-axis shows nominal sampling times referenced to the start of the infusion.

**Figure 3:**
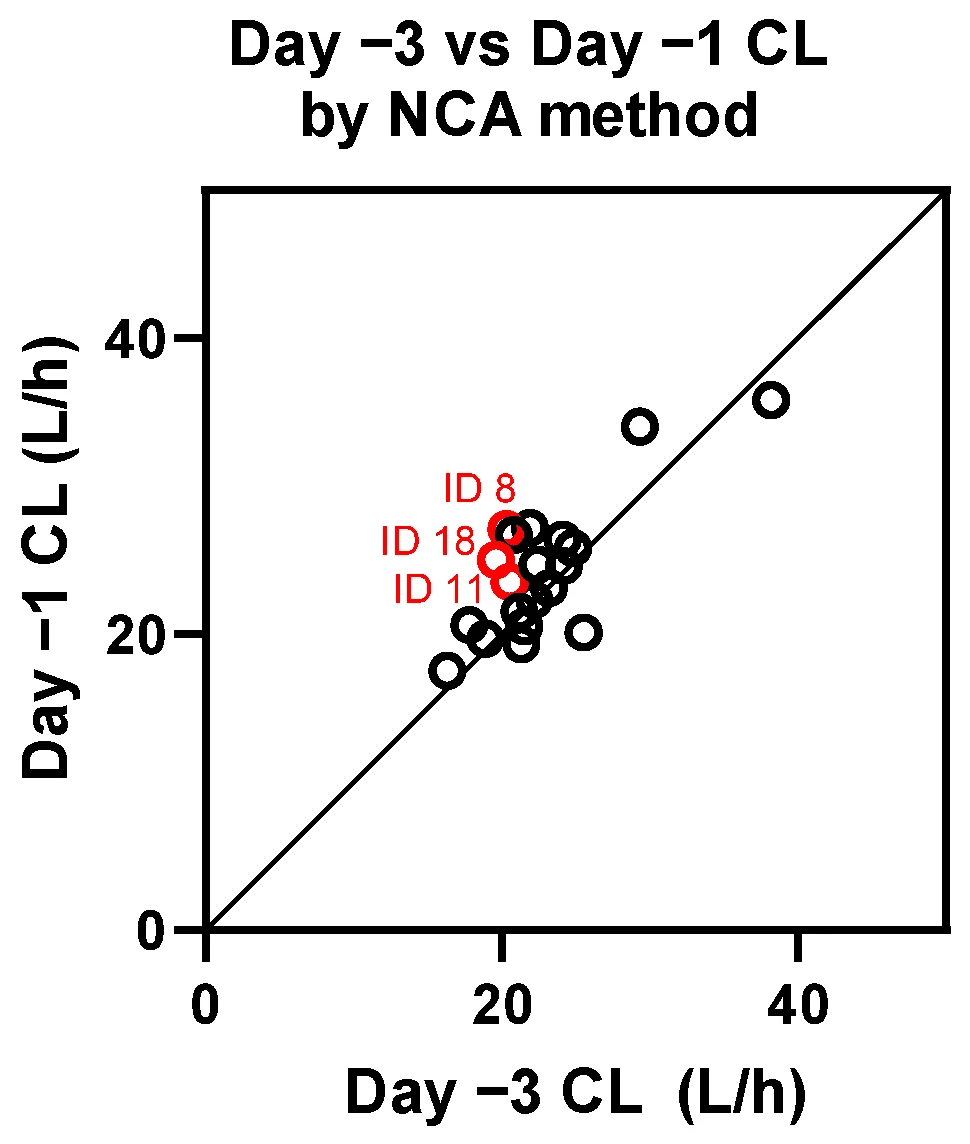
Comparison of melphalan NCA CL estimates between Day −3 and Day −1. Diagonal line represents identity. ID 8 and ID 11 are the patients missing last 2 samples on Day −1; ID 18 is the patient missing last 3 samples on Day −1.

Two hundred seventy (270) of 273 samples (99%) were used in the NCA estimation after exclusion of three outliers. The outliers were identified by visual inspection of the concentration–time profiles because their concentrations were inconsistent with the expected concentration–time profile and with adjacent sampling time points (Figure S1). Melphalan concentration–time profiles on Day −3 and Day −1 by cohort are summarized in Figure 2. Using NCA, the median Day −3 CL was 22.1 (range: 16.3–38.2) L/h in Cohort 1 and 21.4 (range: 17.8–25.5) L/h in Cohort 2. Median Day −1 CL was 26.3 (range: 17.5–35.8) L/h and 22.5 (range: 19.7–26.4) L/h in Cohort 1 and 2, respectively. The intra-individual percentage difference in CL between Day −3 and Day −1 ranged from −21% to 33% among the 20 patients (Figure 3). For the three patients with missing samples on Day −1, the Day −1 CL estimates were 33% (ID 8), 15% (ID 11), and 27% (ID 18) higher than their corresponding Day −3 CL estimates. These higher Day −1 CL values were attributed to missing Day −1 samples in the terminal phase needed to appropriately estimate the elimination rate constant when using NCA. In these cases, the percentage of AUC extrapolated using the NCA method was 19.0% for ID 8, 28.4% for ID 11, and 31.5% for ID 18, close to or exceeding the 20% threshold typically considered acceptable for reliable NCA estimates [35]. Excluding these three patients, Day −1 CL was not significantly different from the Day −3 CL (*n* = 17, *p* = 0.1423 by Wilcoxon signed-rank test).

Using Day −3 NCA results, Day −1 doses were determined for all patients, with dose reductions from Day −3 to Day −1 occurring in 19 (95%) of 20 patients. As a result, the total optimized melphalan dose (Day −3 + Day −1) was a median of 144 (range: 116–196) mg/m^2^ in Cohort 1 and 150 (range: 137–203) mg/m^2^ in Cohort 2. This was 28% and 25% less than the standard HDM dose of 200 mg/m^2^ for Cohorts 1 and 2, respectively.

### 3.4. AUC-Targeting Performance Using NCA

**Figure 4:**
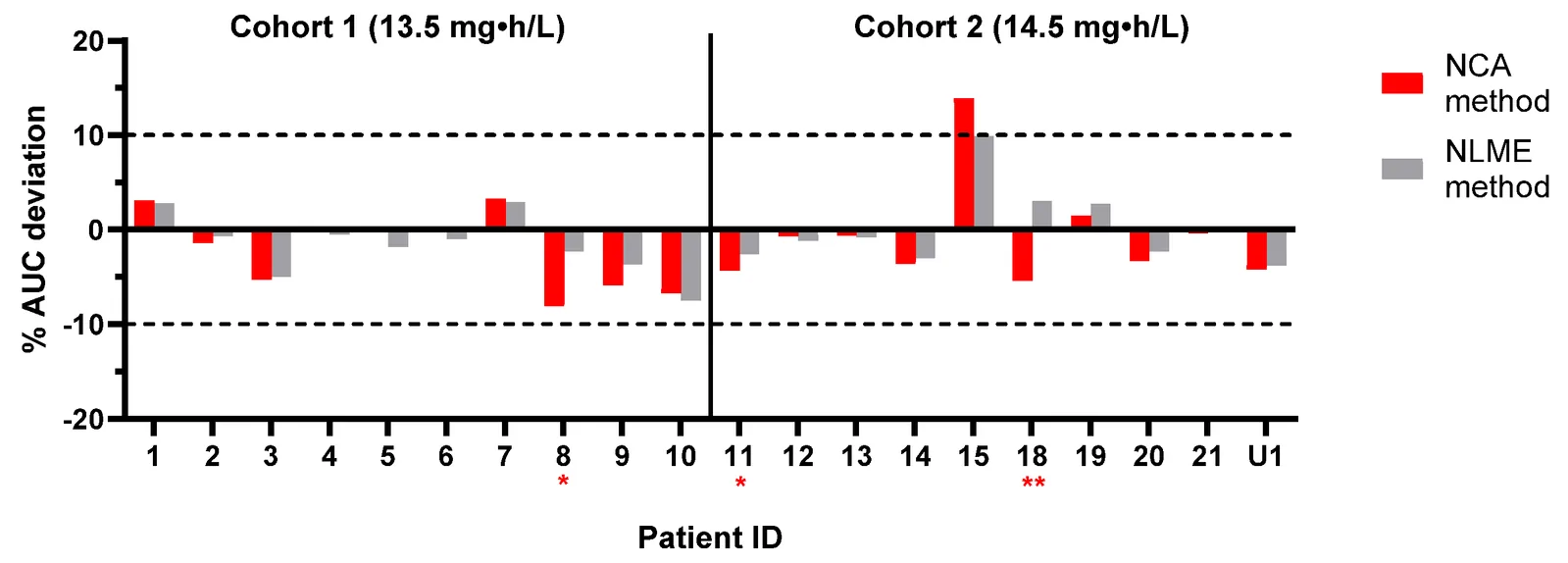
Percent deviation from target AUC using NCA method (red) and NLME method (gray). % AUC deviation by NCA method was calculated as $\%AUC\;deviation=({AUC}_{achieved}-{AUC}_{target})/{AUC}_{target}\times 100$, where ${AUC}_{target}$ was 13.5 mg × h/L in Cohort 1 and 14.5 mg × h/L in Cohort 2. For the NCA method, ${AUC}_{achieved}$ represents the observed total AUC estimated by NCA using actual Day −3 and Day −1 doses. For the NLME method, ${AUC}_{achieved}$ represents the total AUC derived under the NLME approach, calculated as the sum of the NLME-estimated Day −3 AUC and the Day −1 AUC calculated using the NLME-derived Day −1 dose. Dashed lines represent ±10% deviation from the target. * ID 8 and ID 11 are the patients missing last 2 samples on Day −1; ** ID 18 is the patient missing last 3 samples on Day −1.

In cohort 1 with AUC target 13.5 mg × h/L, the median total AUC was 13.4 mg × h/L (CV = 4.20%; range: 12.4–13.9). When the patient with missing Day −1 samples (ID 8) was excluded, the median total AUC was 13.5 mg × h/L (CV = 3.80%; range: 12.6–13.9). In Cohort 2 with AUC target 14.5 mg × h/L, the median total AUC was 14.2 mg × h/L (CV = 5.62%; range: 13.7–16.5). When the two patients with missing samples on Day −1 (ID 11 and 18) were excluded, the median total AUC was 14.4 mg × h/L (CV = 5.81%; range: 13.9–16.5). Ultimately, all patients achieved AUCs within −8.1% to +13.9% of the target AUC, and 19 of 20 patients achieved total AUC within ± 10% of the targets (Figure 4, Tables S4 and S5). In addition to our success in hitting discrete AUC targets, and as our clinical protocol defined AUC target windows that were arbitrarily set as one unit wide (i.e., 13–14 and 14.1–15 mg × h/L, respectively for Cohorts 1 and 2), we further assessed the success of our approach in achieving these narrow AUC target windows. Please see the supplemental materials for results of these analyses (Supplementary Materials Results, Figure S2, Tables S4 and S5).

**Figure 5:**
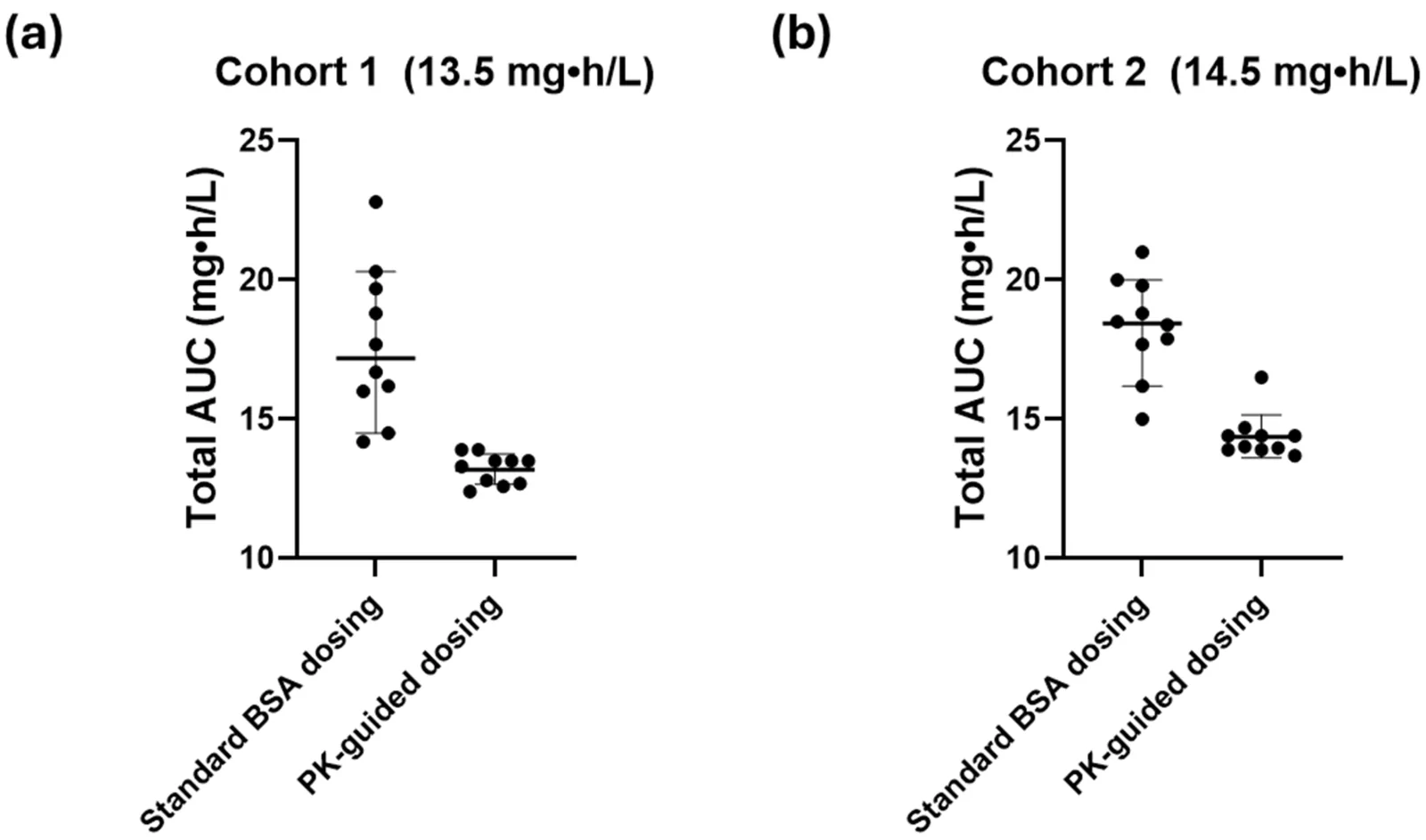
Comparison of total melphalan AUC achieved under standard BSA-based dosing versus PK-guided (NCA-based) dosing in (**a**) Cohort 1 (target 13.5 mg × h/L) and (**b**) Cohort 2 (target 14.5 mg × h/L). Under standard BSA-based dosing, total AUC was simulated assuming administration of 200 mg/m^2^ (100 mg/m^2^ on Day −3 and Day −1) using NCA-estimated CL from each respective day. PK-guided dosing reflects the observed total AUC following 100 mg/m^2^ on Day −3 and an adjusted Day −1 dose. Each dot represents an individual patient. Data are shown as median ± 95% CI.

We compared the observed total AUCs under PK-guided dosing with those that would have resulted from the standard BSA-based dosing. Simulated total AUC under 200 mg/m^2^ dosing had a median of 17.2 mg × h/L (CV = 15.34%, range: 14.2–22.8 mg × h/L) in Cohort 1 and 18.5 mg × h/L (CV = 9.74%, range: 15.0–21.0 mg × h/L) in Cohort 2. In contrast, our PK-guided dosing significantly reduced the AUC variability in both cohorts (*p* < 0.05 for both cohorts by F-test) (Figure 5).

### 3.5. AUC-Targeting Performance Using NLME

**Figure 6:**
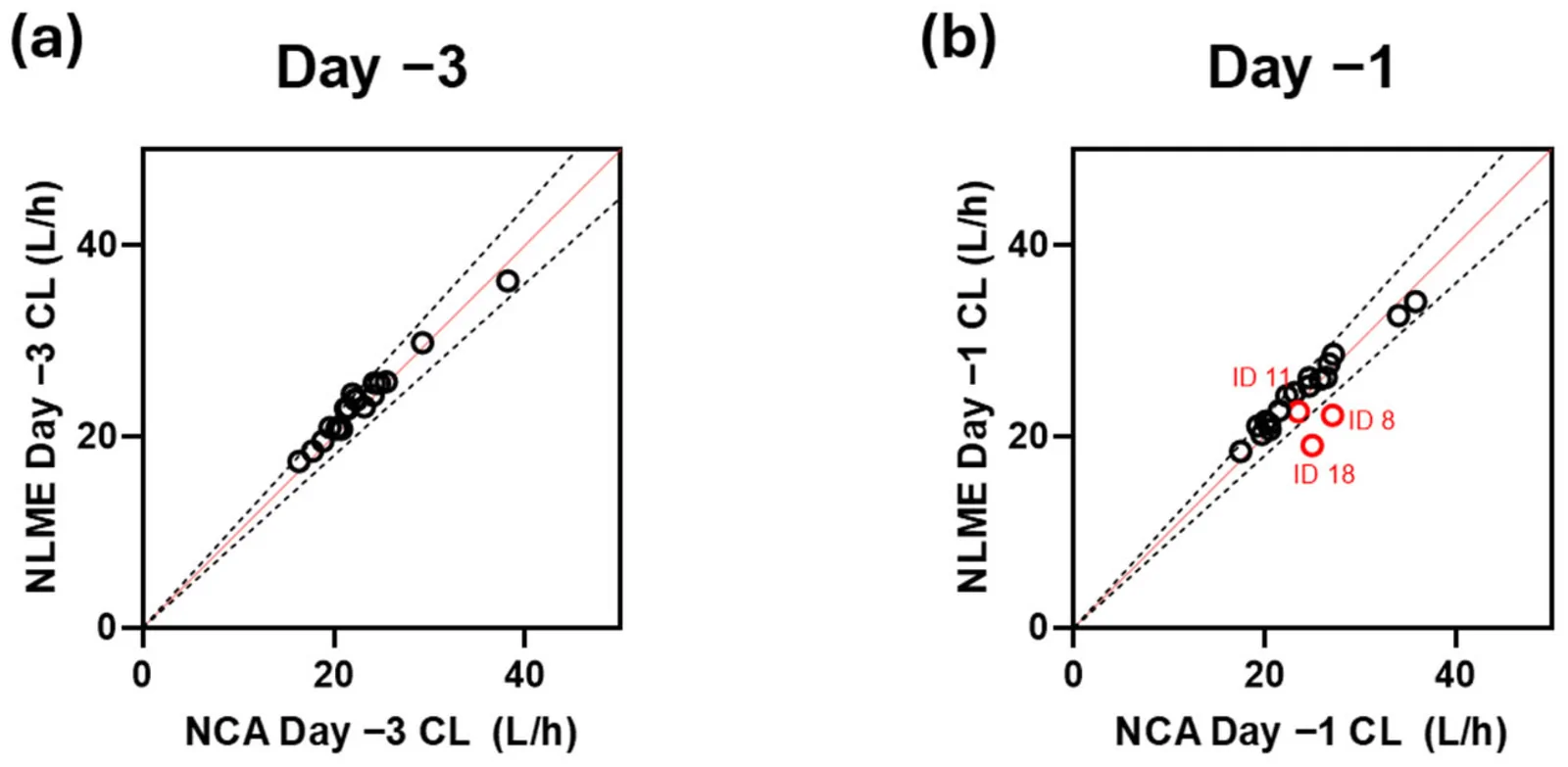
Comparison of melphalan CL estimates between NLME and NCA methods on (**a**) Day −3 and (**b**) Day −1. The solid red line represents the line of identity and the dashed black lines indicate ± 10% of the identity line. Each circle represents an individual patient. Patients with missing samples on Day −1 (ID 8, 11, and 18) are highlighted in red.

A post hoc analysis was performed to retrospectively compare the NLME method with the implemented NCA method in individual CL estimates AUC target attainments. Overall, CL estimated by the NLME method was similar to that obtained by NCA. On Day −3, NLME-estimated CL closely matched NCA results, with all CL falling within ±12% (range: −5.0–11.4%) of the NCA-estimated CL (Figure 6a). On Day −1, NLME-estimated CL was within −23.6% to 9.5% of the NCA-estimated CL (Figure 6b). Larger discrepancies were observed in the three patients (ID 8, 11, and 18) who had missing terminal samples on Day −1, resulting in differences of −17.9%, −3.9%, and −23.6%, respectively. In these patients, NCA estimates were unreliable due to incomplete terminal-phase sampling, whereas NLME-estimated CL values were consistent with Day −3 estimates and appeared physiologically reasonable despite incomplete sampling. When these three patients were excluded, Day −1 NLME-estimated CL was within ±10% (range: −4.8–9.5%) of the NCA estimates.

We next evaluated AUC targeting performance when using NLME to guide Day −1 dose recommendations. For each patient, the post hoc NLME-derived total dose (i.e., sum of the actual Day −3 dose and the NLME-derived Day −1 dose) and total AUC (${Total\;AUC}_{NLME}$) were calculated. The median post hoc NLME-derived total dose was 150 mg/m^2^ (range: 122–186 mg/m^2^) in Cohort 1 and 157 mg/m^2^ (range: 147–205 mg/m^2^) in Cohort 2. This deviated from the standard 200 mg/m^2^ dose by −25% (range: −39.1% to −7.0%) and −22% (range: −26.4% to 2.4%), respectively. In Cohort 1, the median ${Total\;AUC}_{NLME}$ was 13.3 mg × h/L (CV = 3.28%; range: 12.5–13.9 mg × h/L), and in Cohort 2 it was 14.4 mg × h/L (CV = 4.09%; range: 14.0–15.9 mg × h/L). Overall, the post hoc NLME-based dosing showed improved performance with 13 (65%) of 20 patients achieving AUCs closer to the target AUC, and all 20 patients falling within ±10% of the target AUC (range: −7.5–9.9%) when compared to the wider deviation observed with the NCA-based dosing (range: −8.1–13.9%) (Figure 4).

### 3.6. Performance of Sparse Sampling

**Figure 7:**
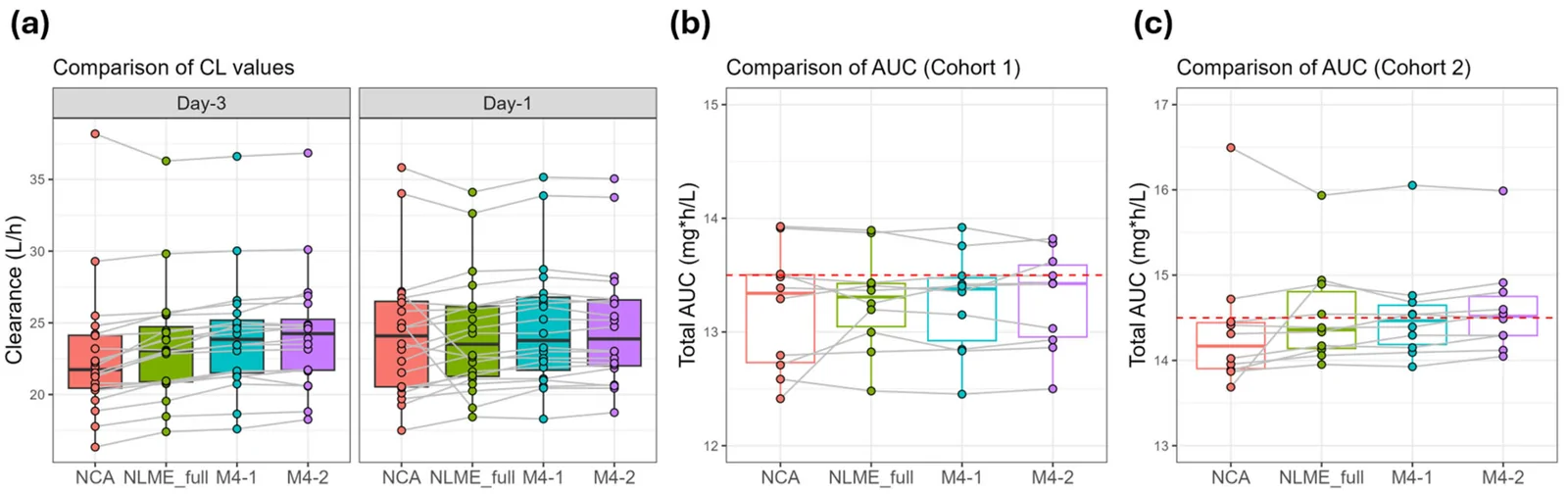
Comparisons of (**a**) CL estimates on Day −3 and Day −1, and total AUCs in (**b**) Cohort 1 and (**c**) Cohort 2 across different methods: NCA (red), NLME using the original sampling scheme (NLME_full, green), NLME using the M4-1 (blue), and NLME using the M4-2 (purple). Red dashed lines in (**b**,**c**) indicate the respective AUC targets. Each point represents an individual patient; lines connect paired estimates across methods.

The impact of reduced sampling from seven to four time points was evaluated using observed PK data from all 20 Phase A patients. The two four-sample test designs included end of the infusion (0.5), 0.75, 1.75, and 4.5 h after start of infusion for the M4-1 design, and end of the infusion (0.5), 0.75, 1.75, and 3 h after start of infusion for the M4-2 design. Post hoc CL estimates and AUC-targeting performance obtained using these test designs were compared to those obtained using the original sampling scheme with either the NLME method (NLME_full) or the NCA method (NCA) (Figure 7). CL values estimated by either sparse design were within ±10% of those estimated by NLME_full. Generally, the distributions of CL estimates from M4-1 and M4-2 were similar to those from NCA and NLME_full. AUC targeting performance was also preserved with the sparse sampling designs, as the distributions of predicted total AUCs using the sparse designs were similar to those obtained using the full seven-sample design. Using the M4-1, the median ${Total\;AUC}_{NLME}$ was 13.4 mg × h/L (CV = 3.38%; range: 12.5–13.9 mg × h/L) in Cohort 1, and 14.5 mg × h/L (CV = 4.08%; range: 13.9–16.1 mg × h/L) in Cohort 2. Using the M4-2, the median ${Total\;AUC}_{NLME}$ was 13.4 mg × h/L (CV = 3.28%; range: 12.5–13.8 mg × h/L) in Cohort 1, and 14.5 mg × h/L (CV = 3.82%; range: 14.0–16.0 mg × h/L) in Cohort 2.

In summary, the AUC-targeting performance of NLME methods utilizing four PK samples was comparable to the performance achieved using the full PK profile with seven samples. These findings support the implementation of a four-sample sparse sampling design for Phase B of the MyMel study.

## 4. Discussion

Standard BSA-based melphalan dosing has been reported to result in up to five-fold variation in systemic exposure, as measured by AUC, and this variability has been associated with clinical outcomes in several studies [11,12,14,15,16,17,18,19]. Unfortunately, the current BSA-based HDM dosing fails to account for variables that can alter AUC, and this may contribute to variability in treatment outcomes.

Prospective PK-guided melphalan dosing strategies have been explored, but not all approaches have been successful. A pre-transplant test dose of 10 mg/m^2^ was unreliable in predicting the full dose needed to achieve a target AUC of 13.5 ± 1 mg × h/L [28]. On the other hand, Nath et al., 2022 used a 20 mg/m^2^ test dose to predict the full dose to achieve a target AUC of 12.8 mg × h/L ± 15% (10.9–14.7 mg × h/L) in adult MM patients and achieved an 80% (4 out of 5) attainment rate [30]. Shah et al., 2019 administered 100 mg/m^2^ propylene glycol-free melphalan 2 days prior to ASCT and individualized dose on Day −1 to attain a target total AUC of 13.5 mg × h/L ± 1, with 87% (13 of 15 patients) achieving the target [29]. While these more recent results were encouraging, the number of MM patients was small (five in Nath et al. and 11 of 15 patients in Shah et al. received actual PK-guided dosing), and real-time dose adjustment occurred at a single center in Shah et al. Therefore, the impact of multi-institution coordination and sample shipping on sample quality and PK results remains uncertain. More importantly, an optimal AUC target balancing efficacy and toxicity has yet to be determined.

Although clinical variables such as renal function, hematocrit, and fat-free mass influence melphalan AUC, a priori dose adjustment using a population PK model without individual PK observations was insufficient to reliably achieve target AUCs [31]. Our previously developed population PK model for melphalan only explained 18% of the observed interpatient PK variability, further highlighting the limitations of model-informed dosing without real-time concentration–time data. Accordingly, we implemented a real-time PK-guided dosing strategy within a multi-center Phase 1 trial to evaluate the feasibility and accuracy of target AUC attainment and to ultimately identify the optimal AUC range based on efficacy and toxicity. Here, we reported the final results of the Phase A portion of this trial (*n* = 20).

Phase A demonstrated the ability to achieve a predefined AUC target for HDM using a split dose approach: 100 mg/m^2^ on Day −3 relative to transplant, followed by an adjusted dose on Day −1 based on the observed AUC from Day −3. To support this strategy, we developed a clinical workflow that enables real-time melphalan therapeutic drug monitoring in a real-world setting. Unlike drugs that are administered repeatedly over time, HDM is given as a one-time conditioning regimen prior to ASCT. This limited dosing window necessitates a real-time workflow and assay with rapid turnaround to enable dose modification based on an individual’s PK parameters. Our workflow enabled rapid and end-to-end execution across clinical and laboratory settings, including patient blood draws, same-day sample processing and overnight shipping, rapid bioanalysis using a validated LC-MS/MS assay, and timely reporting of PK results to guide individualized dose adjustment.

For this phase, NCA was used as the primary method for real-time dose adjustment based on FDA recommendations. We showed that compared to what would have occurred with standard BSA dosing, PK-guided dose adjustment resulted in significantly less AUC variability. 19 of 20 patients deviated from their respective target (13.5 or 14.5 mg × h/L) by less than 10% using the NCA-based PK-guided dosing. One remaining patient deviated by 13.9% from the target and exhibited a relatively large change in CL between Day −3 and Day −1, with a decrease of over 20%. However, with the available data, we could not determine the cause for this change in CL over the dosing period.

Interestingly, we noticed that the variability observed in our simulated BSA-based AUC was smaller than the up to five-fold variability reported in prior studies. This discrepancy likely reflects differences in study design and patient population. Previous reports included heterogeneous patient populations and broad dose ranges (e.g., 89 to 216 mg/m^2^ in Nath et al., 2016) with routine dose modification for renal dysfunction or obesity [11,14,15,18,19]. In contrast, our simulated BSA-based comparison assumed fixed 200 mg/m^2^ dosing (100 mg/m^2^ on Days −3 and −1) within a protocol-defined population excluding patients with eGFR < 40 mL/min. Creatinine clearance (CrCL) in our population ranged from 53.4 to 188 mL/min (3.5-fold), which is narrower than CrCL distributions reported in the prior studies (e.g., 14–255 mL/min in Nath et al., 2016; 0.2–196.5 mL/min in Cho et al., 2017) [11,15]. Given the known effect of renal function on melphalan CL, this more homogenous population in our study likely contributed to the smaller AUC range observed in our simulation.

Our PK-guided dosing resulted in a reduction in total melphalan dose compared to 200 mg/m^2^ dosing, with a median dose reduction of approximately 22–25% in both cohorts. This suggests that fixed 200 mg/m^2^ dosing would have produced exposures exceeding the Phase A targets in this patient population. The Phase A targets were selected to start at exposure levels slightly above the previously reported median AUC associated with improved overall survival [15], which fall within the lower-to-mid range of historically observed exposures across broader dosing regimens [11,15,19]. Differences from historical exposure values may reflect differences in patient characteristics, dosing practices, and study methodologies across studies.

While NCA is a straightforward method for estimating PK parameters, it is sensitive to sampling variability, does not account for covariates, and ignores interindividual random effects. We therefore utilized our melphalan NLME model to perform post hoc estimation of CL and AUC for each individual as a direct comparison and ‘quality check’ with NCA estimates. NLME offers several benefits over NCA for PK parameter estimation: (1) it leverages prior knowledge of melphalan PK behavior in this patient population, reducing dependence on individual data points and providing stable CL estimates [36]; (2) it is more robust to missing PK samples, maintaining estimation accuracy despite incomplete data; (3) these features enable the use of a sparse sampling design, reducing clinical and financial burdens; (4) NLME allows integration of clinically relevant covariates such as creatinine clearance, hematocrit, and fat-free mass, supporting further investigation into sources of PK variability and enabling more precise individualized dosing. With the increasing adoption of model-informed precision dosing platforms in clinical practice, we explored the feasibility of NLME-based dose adjustment in the post hoc analysis. Although NLME was not used prospectively for dose determination in Phase A, NLME-derived Day −1 doses were available before Day −1 for all patients. The web-based MyMel Shiny App is being expanded to incorporate NLME-based estimation and automated dose recommendations, which may facilitate implementation of the real-time NLME-based dose adjustment across multiple institutions in future phases.

Across both AUC cohorts, NLME and NCA resulted in generally similar CL estimates for both Day −3 and Day −1, with NLME estimates deviating from NCA by less than ±12% for the majority of patients. The NLME method would have brought 13 (65%) of 20 patients closer to their AUC target, thereby resulting in all 20 patients achieving total AUCs within ±10% of their targets (−7.5–9.9%). Notably, NLME demonstrated the most benefit in cases of missing PK data. For the three patients missing the last two or three samples on Day −1, the percentage of AUC extrapolated using the NCA method ranged from 19.0% to 31.5%, approaching or exceeding the commonly accepted threshold above which NCA reliability is generally considered poor. In these patients, the Day −1 NCA-estimated CL differed from the corresponding Day −3 NCA-estimated CL by up to 33%, suggesting that the NCA estimates were sensitive to the missing terminal samples. In contrast, the NLME-estimated CL remained stable between Day −3 and Day −1 and was more physiologically plausible. Finally, we identified sparse sampling designs that maintain performance of the NLME-guided dosing. The two identified sampling designs (M4-1 and M4-2) consisting of four time points preserved accuracy in estimating CL and AUC. Post hoc analysis showed that CL estimates from these sampling schemes were within ±10% of the estimates using the original full sampling scheme (seven samples). In addition, AUC-targeting performance was comparable. These findings support the feasibility of sparse sampling designs for Phase B of the MyMel study to reduce patient burden while maintaining dose individualization accuracy.

We acknowledge that Phase A included a relatively small sample size (*n* = 20) and a more homogenous population due to the enrollment criteria requiring preserved renal function. Phase B will expand eligibility to include patients with renal dysfunction and those receiving HDM-ASCT in the first-line setting. Seven additional escalating AUC target cohorts, along with a planned 20-patient expansion cohort at the MTSE, will provide a larger and more heterogenous population for exposure-response evaluation. Notably, 45% of patients in Phase A were Black, providing representation not extensively reported in prior melphalan PK studies. Future analyses may reveal whether demographic factors contribute to variability in melphalan PK. Completion of the MyMel study will provide a substantially larger dataset for refinement of the published population PK model, evaluation of additional covariates, and development of an updated model to further support model-informed precision dosing of melphalan.

## 5. Conclusions

In conclusion, we demonstrated the feasibility of prospectively targeting melphalan AUC with high accuracy and rapid turnaround of PK results in a multi-center setting. Post hoc evaluation demonstrated that NLME-based dose adjustment can further improve the target AUC attainment, particularly in the setting of incomplete sampling. Given this, Phase B of this trial will proceed to identify the MTSE of up to seven additional AUC cohorts in a 5 + 5 design using our NLME model and a reduced four-time-point sampling schedule.

## Figures and Tables

**Table 1:** Baseline characteristics of 20 patients in MyMel study Phase A.

Characteristics	Median (Range)
Age (years)	64 (49–75)
BSA (m^2^)	2.06 (1.76–2.63)
Creatinine clearance (mL/min)	97.6 (53.4–188)
Fat-free mass (kg)	61.9 (47.0–87.2)
Hematocrit (%)	35.8 (27.4–43.0)
Height (cm)	174 (155–198)
Median weight (kg)	91.7 (65.0–142)
**Gender**	**Number of Patients (%)**
Male	14 (70)
Female	6 (30)
**Race**	**Number of Patients (%)**
White	10 (50)
Black	9 (45)
Hispanic	1 (5)

## Data Availability

Data presented in this study is contained within the article and Supplementary Materials. Further inquiries can be directed to the corresponding author.

## Supplementary Materials

The following supporting information can be downloaded at https://www.mdpi.com/article/10.3390/pharmaceutics18080924/s1. Figure S1: Individual plasma melphalan concentration–time profiles on (a) Day −3 and (b) Day −1; Figure S2: Target attainment in (a) Cohort 1 (13–14 mg × h/L) and (b) Cohort 2 (14.1–15 mg × h/L), as assessed by NCA on Day −3 (gray) and Day −1 (orange); Table S1: Summary of prior studies evaluating PK-guided melphalan dose individualization; Table S2: Summary of Phase A and B of Phase 1 clinical trial (NCT04483206); Table S3: Primary endpoints of the Phase 1 clinical trial; Table S4: Optimized dosing and resulting AUCs in Cohort 1 based on NCA; Table S5: Optimized dosing and resulting AUCs in Cohort 2 based on NCA; Table S6: Optimization results under different sampling window constraints.

## Abbreviations

The following abbreviations are used in this manuscript:

PK	pharmacokinetics
HDM	high-dose melphalan
ASCT	autologous stem cell transplant
MM	multiple myeloma
BSA	body surface area
AUC	area under the plasma drug concentration–time curve
LC-MS/MS	liquid chromatography-tandem mass spectrometry
NCA	noncompartmental analysis
NLME	nonlinear mixed effects
PFS	progression-free survival
CR	complete remission
MRD	minimal residual disease
IMWG	International Myeloma Working Group
MTSE	maximum tolerated systemic exposure
IRB	institutional review board
FDA	Food and Drug Administration
UIC	University of Illinois Chicago
OSU	The Ohio State University
eGFR	estimated glomerular filtration rate
PhASR	OSU Pharmacoanalytical Shared Resource
CL	clearance
PI	principal investigator
FOCEI	first-order conditional estimation with interaction
CV	coefficient of variation
CI	confidence interval
CrCL	creatinine clearance
