# Supplementary material for: Accuracy of Real-Time PK-Guided Melphalan Dosing in Achieving Target Exposure in Myeloma Patients Undergoing Autologous Transplant
Source: Pharmaceutics. 2026 Jul 27;18(8):924. doi: 10.3390/pharmaceutics18080924 (PMC13516206; doi:10.3390/pharmaceutics18080924)
Supplement: Supplementary file 1 [file pharmaceutics-18-00924-s001.zip › pharmaceutics-4362623-supplementary.pdf]

pharmaceutics

Supplementary Methods

Sparse Sampling Design Optimization

Sparse sampling designs for melphalan PK were optimized using $DESIGN option in NONMEM, version 7.5.0. Sampling windows were constrained such that the earliest allowable time point corresponded to the end of the infusion (0.5 hours after infusion start), while the latest allowable time points were bounded at 4.5, 4.0, 3.5, or 3.0 hours after infusion start, depending on the scenario. A Nelder search was performed to determine the ideal time points for three different cases where the numbers of samples were 7, 5, and 4. A subject number size of 20 was used and specified with GROUPSIZE in the $DESIGN record. Optimized designs were assessed by comparing the negative logarithm of the determinant of the Fisher information matrix (−log(det(FIM)) and the expected standard errors (SE) of PK parameter estimates.

To assess how the optimized designs would perform using real PK data, the observed PK data from all 20 Phase A patients were used. From the original seven PK time points (0.5, 0.58, 0.75, 1, 1.75, 3, 4.5 hours after infusion start), four time points closest to the optimized time points were selected to construct two test sparse sampling designs. These designs consisted of samples at 0.50, 0.75, 1.75, and 4.5 hours after infusion start for M4-1 and at 0.50, 0.75, 1.75, and 3 hours for M4-2. Sparse sampling datasets were created by filtering the observed data to include only the time points in each test design. These datasets were then used to estimate individual PK parameters via post hoc NLME analysis, providing CL, predicted Day −1 AUC, and predicted total AUC to assess the performance of the sparse sampling designs compared to the full seven-sample profile.

Supplementary Results

AUC Target Attainment Within Narrow Prespecified Windows using NCA

Our clinical protocol defined target AUC windows that were arbitrarily set as one unit wide (i.e., 13–14 and 14.1–15 mg×h/L for Cohorts 1 and 2, respectively). We recognized early in this trial that these prespecified AUC windows were very narrow (i.e., 13.5 mg×h/L ± 3.7% and 14.5 mg×h/L ± 3.5%, respectively). Despite these tight boundaries, we were able to demonstrate success of our real-time personalized HDM strategy.

In cohort 1 **(Figure S2** and **Table S4),** 6 of 10 patients achieved the target AUC window of 13–14 mg×h/L, and a median total AUC was 13.4 mg×h/L (CV = 4.20%). When the patient with missing Day −1 samples (ID 8) was excluded, the median total AUC was 13.5 mg×h/L (CV = 3.80%). In Cohort 2 **(Figure S2** and **Table S5),** 4 of 10 patients achieved the narrow target AUC window of 14.1–15 mg×h/L, with a median total AUC of 14.2 mg×h/L (CV = 5.62%). When the two patients with missing samples on Day −1 (ID 11 and ID 18) were excluded, the median AUC was 14.4 mg×h/L (CV = 5.81%).

Among patients who did not fall within the target windows, deviations were generally small. Across both cohorts, 9 (90%) of 10 deviations were below the target window, with a median deviation of 5.3% from the midpoint of the window (13.5 in Cohort 1 and 14.5 in Cohort 2). Only one patient exceeded the target window by 13.9% relative to the midpoint. Overall, all patients achieved total AUCs within −8.1% to +13.9% of the midpoint of their respective target windows.

Based on these findings and operational experience from Phase A, the clinical protocol was amended for Phase B to use discrete AUC targets rather than narrow one-unit-wide windows.

AUC Target Attainment Within Narrow Prespecified Windows using NLME

Of the patients who did not fall within their AUC target windows using the NCA method (four in Cohort 1 and six in Cohort 2), the NLME method would have brought two of four (50%) and three of six (50%) into their respective target windows. Among the five patients who still fell outside the target AUC windows with the NLME method, the deviations from the midpoint of the target window were all within ±10% (range: −6.6% – 9.7%). Importantly, two of the three patients who had missing Day −1 samples were moved within or close to the target window when NLME was used, highlighting how the NLME method is less vulnerable to missing samples when estimating CL.

Optimization Results for Sparse Sampling Designs

Results of the sparse sampling design optimization are summarized in **Table S6**. For all candidate designs evaluated (seven-, five-, and four-sample schemes), the optimization consistently converged to four distinct sampling times, although the specific times differed depending on the constraint on the latest allowable sampling time. This result indicated that only four unique time positions contributed meaningful information within the constrained sampling window. These four points were the boundary positions (constrained minimum and maximum values for times), and two additional time points near 0.7 h and 1.5 h after the start of infusion, with some small variations among the replicates.

With the sampling schemes using four time points, the relative standard errors (RSEs) for the between-subject variabilities of V1 and Q were expectedly high (43.1 – 62.4% for var(V1); 73.1 – 96.1% for var(Q)). However, RSEs for population parameters and for the between-subject variability of CL (var(CL)) remained comparable to those estimated with the full PK profile with seven sampling time points.

Table S1. Summary of prior studies evaluating PK-guided melphalan dose individualization.

Reference	Center	Disease	No. Pts treated	No. Pts who received actual PK-guided dosing	Method	Target AUC	Result
Shah et al., 2017 [Ref 28]	Memorial Sloan Kettering Cancer Center	MM and AL	10 adults (7 MM, 3 AL)	7 adults[a]	10 mg/m^2 test dose of propylene glycol free melphalan (PGF-MEL) was administered within 14 days of ASCT. PK samples were collected at 5, 15, 30, 40, 75, and 150 min after dose, and Day −2 dose was determined and administered.	13.5 mg×h/L ± 1	• Median target dose AUC of the 10 patients was 15.65 mg×h/L (range 10.1–17.2).<br>• No patients receiving the individualized target dose achieved the target AUC.
Shah et al., 2019 [Ref 29]	Memorial Sloan Kettering Cancer Center	MM and AL	15 adults (11 MM, 4 AL)	11 adults[b]	100 mg/m^2 of PGF-MEL was administered on Day −2. PK samples were collected at 5, 15, 30, 40, 75, and 150 min after dose, and Day −1 dose was determined and administered.	13.5 mg×h/L ± 1	• Median total AUC of the 15 patients was 13.2 mg×h/L (range 9.6–15.2).<br>• 13 of 15 patients (87%) achieved the target AUC.

Nath et al., 2022 [Ref 30]	6 centers in Australia	MM	44 adults	5 adults[c]	20 mg/m² test dose[d] was administered 1–3 days prior to HDM administration and autograft. PK samples were collected at 5, 15, 30, 40, 75 and 150 min post-infusion completion, and Day −1 dose was determined. Only 5 of them received actual PK-adjusted dose and others received the remaining 180 mg/m².	12.8 mg×h/L ± 15% (10.9–14.7 mg×h/L)	• Median total AUC of the 5 patients who received the actual PK-guide dosing was 12.6 mg×h/L (range 10.9–17.0).<br>• 4 of 5 patients (80%) achieved the target AUC.
Hughes et al., 2024 [Ref 31]	Memorial Sloan Kettering Cancer Center	Relapsed lymphomas	16 adults	16 adults	> A priori individualized dose of melphalan was predicted and administered using a previously developed population PK model. Carmustine, etoposide, and cytarabine were administered as standard of care.	8.5 mg×h/L ± 1.5	• Median AUC was 9.2 (range 6.2–13.3).<br>• 8 of 16 patients (50%) achieved the target AUC.

[a] Three patients did not receive the PK-guided dosing due to inability to calculate a test dose AUC, toxicity after the test dose, and target dose outside the study safety limit.

[b] Three patients received the capped dose of 220 mg/m². One patient received the minimum 140 mg/m².

[c] Four patients who received a test dose of 10 mg/m² were excluded due to difficulties in detecting low melphalan concentrations. PK results for the 20 mg/m² test dose were available before HDM dosing for 31 patients. Of the 31 patients, 26 patients received the remaining 180 mg/m² by physicians' decisions.

[d] The first four of the 44 patients received a test dose of 10 mg/m².

AL; AL Amyloidosis

Table S2. Summary of Phases A and B of Phase 1 clinical trial (NCT04483206).

Phase A			Phase B	
Eligibility:	2+ lines of therapy<br>Eligible for 200 mg/m² normally<br>eGFR≥40 ml/min	FDA Meeting	Eligibility:	1+ lines of therapy<br>any eGFR
PK-guided dosing	Noncompartmental analysis (NCA)		PK-guided dosing	Nonlinear mixed effects modeling (NLME)
Pharmacokinetic assessments:	Day -3<br>Day -1		Pharmacokinetic assessments:	Day -3<br>Day -1

Table S3. Primary endpoints of the Phase 1 clinical trial.

Hypothesis	Objective	Endpoint
A recommended phase 2 systemic exposure level will be identified based on toxicities	Identify safety and preliminary efficacy within each sys-	Measure proportion of patients with clinically significant mucositis[1], tachyarrhth-

and response at three months.	temic exposure range of melphalan using a 5+5 design.	mia[2], delayed engraftment[3], and MRD negativity post-transplant[4].

1. Grade 3–4 oral mucositis using oral mucositis assessment scale (OMAS).
2. Non-sinus tachyarrhythmia with heart rate > 150 beats per minute or with hemodynamic instability.
3. Delayed engraftment is defined as failure to achieve an absolute neutrophil count > 500/uL by Day +20.
4. MRD status per next-generation sequencing at Day +90 in patients with suspected complete response.

Table S4. Optimized dosing and resulting AUCs in Cohort 1 based on NCA.

Target AUC	Location	Patient ID	Total dose (mg/m²)	NCA estimated Total AUC (mg×h/L)	% AUC deviation from the target
13.5	Emory	1	140	13.9	3.1
	Emory	2	163	13.3	−1.4
	Emory	3	137	12.8	−5.3
	Emory	4	136	13.5	−0.1
	Emory	5	143	13.5	0.0
	Emory	6	116	13.5	0.2
	Emory	7	196	13.9	3.3
	Emory	8*	148	12.4	−8.1
	Emory	9	173	12.7	−5.9
	Emory	10	144	12.6	−6.7
		**Median**	144	13.4	−0.8
		**Min**	116	12.4	−8.1
		**Max**	196	13.9	3.3
		**%CV**	14.9	4.20	3.1

*Missing last two time points on Day −1.

Table S5. Optimized dosing and resulting AUCs in Cohort 2 based on NCA.

Target AUC	Location	Patient ID	Total dose (mg/m²)	NCA estimated Total AUC (mg×h/L)	% AUC deviation from the target
14.5	Emory	11*	152	13.9	−4.3
	Emory	12	144	14.4	−0.7
	Emory	13	155	14.4	−0.6
	Emory	14	185	13.97	−3.6
	Emory	15	203	16.5	13.9
	Emory	18**	138	13.7	−5.4
	Emory	19	148	14.7	1.5
	Emory	20	156	14.02	−3.3
	Emory	21	137	14.4	−0.4
	UIC	U1	147	13.9	−4.2
		**Median**	150	14.2	−2.0
		**Min**	137	13.7	−5.4
		**Max**	203	16.5	13.9
		**%CV**	13.5	5.62	−4.3

*Missing last two time points on Day −1.

**Missing last three time points on Day −1.

Table S6. Optimization results under different sampling window constraints.

Number of time points explored		7 samples				5 samples				4 samples			
Description	Current design	Optimized Tlast=4.5	Optimized Tlast=4	Optimized Tlast=3.5	Optimized Tlast=3	Optimized Tlast=4.5	Optimized Tlast=4	Optimized Tlast=3.5	Optimized Tlast=3	Optimized Tlast=4.5	Optimized Tlast=4	Optimized Tlast=3.5	Optimized Tlast=3
−log(det(FIM))	−24.91	−25.824	−25.136	−24.462	−23.652	−23.795	−23.225	−22.526	−21.641	−22.239	−21.632	−20.932	−20.082
OFV diff with the current design	0	−0.914	−0.226	0.448	1.258	1.115	1.685	2.384	3.269	2.671	3.278	3.978	4.828
%RSE(CL)	4.6	4.6	4.6	4.6	4.5	4.6	4.6	4.6	4.6	4.7	4.7	4.7	4.7
%RSE(V1)	11.5	9.5	11.6	11.8	11.2	10.9	11.1	11.2	11.4	12.7	12.9	13.2	13.4
%RSE(Q)	20	17.1	17.7	18.2	22.3	22.3	22.7	23.2	23.8	22.6	23	23.6	24.3
%RSE(V2)	10.2	9.5	10.1	10.3	11.6	11.6	11.7	11.8	12	12.2	12.4	12.6	12.8
%RSE(var(CL))	24.3	24.3	24.3	24.3	24.3	25.5	25.4	25.4	25.2	25.9	25.9	25.8	25.6
%RSE(var(V1))	36.5	35.1	37.3	41.7	43	36.2	39.2	43.1	49.8	43.1	47.7	53.5	62.4
%RSE(var(Q))	55.6	47	50.9	56.3	66.7	61.6	68.8	77.1	86	73.1	82.1	89.6	96.1
%RSE(var(sigma))	11.1	11.2	11.1	11.1	10.8	15.7	15.4	14.9	14.2	21.9	21.5	20.5	18.7
Sample time 1	0.5	0.5000	0.5000	0.5000	0.5000	0.5000	0.5000	0.5000	0.5000	0.5000	0.5000	0.5000	0.5000
Sample time 2	0.58	0.5000	0.7145	0.7058	0.7058	0.5000	0.5000	0.5000	0.5000	0.7191	0.7082	0.6995	0.6954
Sample time 3	0.75	0.7269	0.7149	0.7062	0.7062	0.7322	0.7251	0.7175	0.7103	1.5695	1.5382	1.5101	1.4510
Sample time 4	1	0.7275	1.5344	1.4895	1.4895	1.5704	1.5347	1.4957	1.4489	4.5000	4.0000	3.4999	3.0000
Sample time 5	1.75	1.5601	1.5394	1.4902	1.4902	4.4999	3.9999	3.4999	2.9999				
Sample time 6	3	1.5636	3.9991	3.4997	3.4997								
Sample time 7	4.5	4.5000	3.9996	3.4999	3.4999								

FIM, Fisher information matrix; OFV, objective function value; RSE, relative standard error; var, variations in the parameter; CL, clearance; V1, central volume of distribution; V2, peripheral volume of distribution.

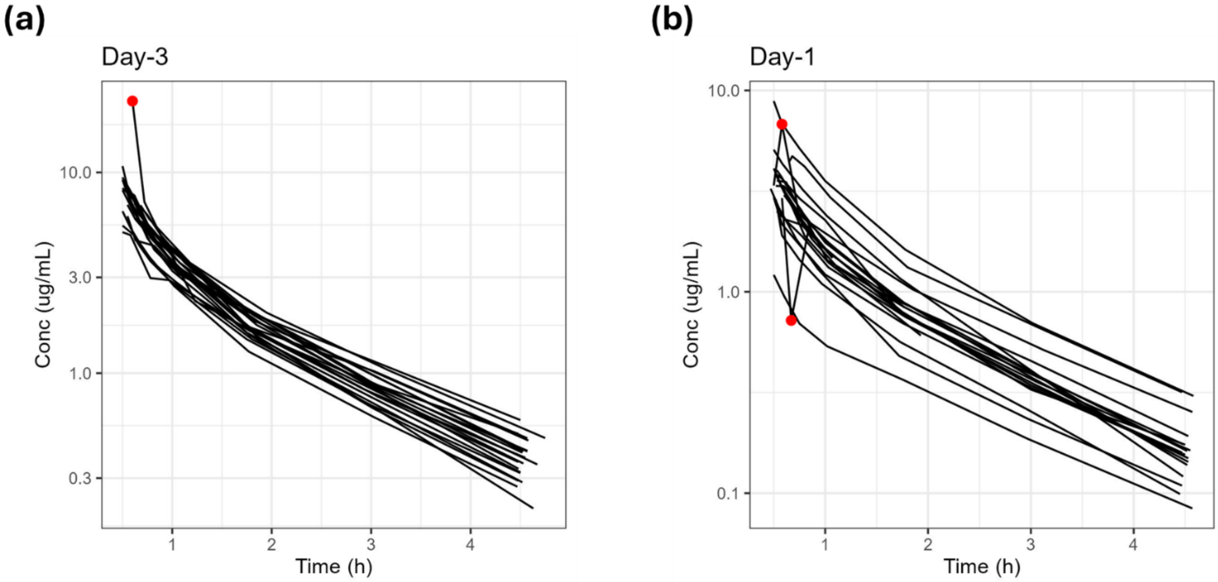

Figure S1. Individual plasma melphalan concentration–time profiles on (a) Day −3 and (b) Day −1. Y-axis on a logarithmic scale. Solid black lines represent concentration–time profiles of each patient and red dots indicate the three concentration observations identified as outliers by visual inspection. Bioanalysis was repeated on these three samples and concentrations were verified.

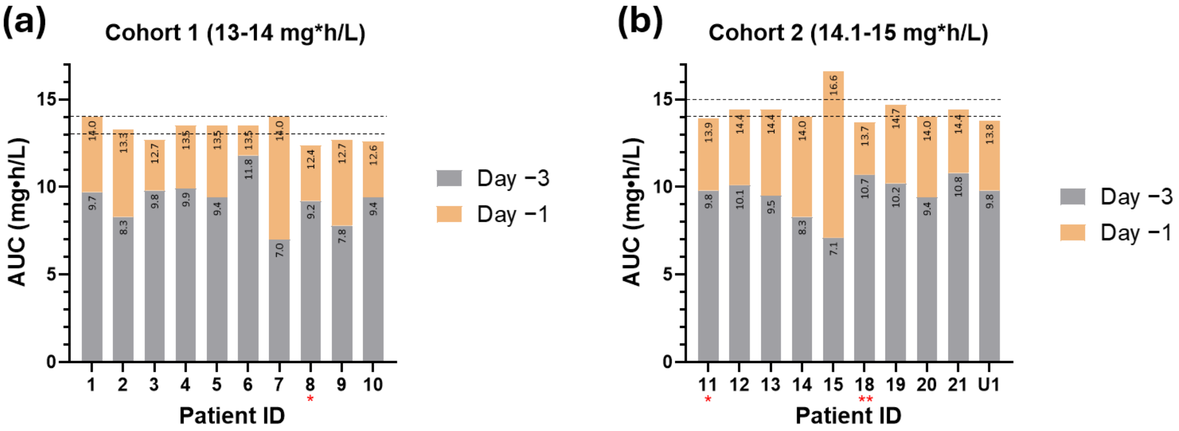

Figure S2. Target attainment in **(a)** Cohort 1 (13–14 mg×h/L) and **(b)** Cohort 2 (14.1–15 mg×h/L), as assessed by NCA on Day −3 (gray) and Day −1 (orange). Dashed lines indicate the respective AUC targets. *Missing last two time points on Day −1. **Missing last three time points on Day −1.
